# Research progress on the ethanol precipitation process of traditional Chinese medicine

**DOI:** 10.1186/s13020-020-00366-2

**Published:** 2020-08-10

**Authors:** Yanni Tai, Jichen Shen, Yu Luo, Haibin Qu, Xingchu Gong

**Affiliations:** Pharmaceutical Informatics Institute, College of Pharmaceutical Sciences, Zhejiang University, Hangzhou, 310058 Zhejiang China

**Keywords:** Ethanol precipitation process, Critical process parameters, Optimization methods, Process modeling, Process monitoring technology

## Abstract

Ethanol precipitation is a purification process widely used in the purification of Chinese medicine concentrates. This article reviews the research progress on the process mechanism of ethanol precipitation, ethanol precipitation process application for bioactive component purification, ethanol precipitation and traditional Chinese medicine quality, ethanol precipitation equipment, critical parameters, parameter research methods, process modeling and calculation methods, and process monitoring technology. This review proposes that ethanol precipitation technology should be further developed in terms of five aspects, namely, an in-depth study of the mechanism, further study of the effects on traditional Chinese medicine quality, improvement of the quality control of concentrates, development of new process detection methods, and development of a complete intelligent set of equipment.

## Background

In the 1950s, a batch of modern dosage forms of traditional Chinese medicine (TCM) appeared in the climax of national dosage form reform, such as tablets, capsules, injections, granules, and mixtures. To meet the higher refining requirements of these dosage forms for the TCM systems, water extraction and ethanol precipitation (EP) technologies have been widely used in the production of TCM. The ethanol precipitation process (EPP) has many advantages, such as simple operation, easy amplification, and solvent safety. The EPP can effectively remove highly polar molecules such as sugars, salts, and proteins, and it is beneficial to reduce the dosage [1]. In the production of TCM injections, multiple EP steps are often used to remove impurities entirely. Alkaline EP can remove tannins and further improve the safety of TCM injections [2]. In the Chinese Pharmacopoeia (2015 Edition) [4], there were 274 kinds of Chinese herbal medicines involving EPP, accounting for 18.4% of the 1493 prescriptions and single formulations [3, 4]. At present, the EPP also has disadvantages, including severe encapsulation loss, the low heat transfer efficiency of equipment, long standing time for precipitation, high energy consumption, and low efficiency of slag removal.

EP is often the first refining process or even the only refining process after the extraction of TCMs. The quality of the EPP has a significant impact on the difficulty of follow-up preparations and the quality of the final drug. In recent years, with the continuous improvement of Chinese medicine standards, the EPP has attracted much attention from academic and industrial circles. This article mainly reviews the research progress on the process mechanism of EPP, EPP application for bioactive component purification, EP and TCM quality, EP equipment, critical parameters, parameter research methods, process modeling and calculation methods, and process monitoring technology and proposes future development directions.

## EPP mechanism

Generally, after adding ethanol to a TCM concentrate, the solubility of some strongly polar components and macromolecular components decreases in the system, causing precipitation. In a mixture of water and ethanol, the solubility of monosaccharides and oligosaccharides such as d-glucose, d-fructose, sucrose, maltose, raffinose, trehalose, and cyclodextrin has been reported [5–8]. Overall, the solubility of sugar components decreases with decreasing temperature or increasing ethanol content, which shows that properly increasing the ethanol concentration of the supernatant and lowering the standing temperature is beneficial to remove more sugar impurities. Bouchard et al. also reported solubility data of polysaccharides of inulin and dextran [5]. Ku et al. verified that polysaccharides with higher degree of polymerization were easier to precipitate in the mixed solvent of ethanol and water [9]. Boulet et al. found protein precipitated at different ratios when pH value varied in the mixture of ethanol and water. More protein precipitated as ethanol content in the mixed solvent increased [10].

There have been many studies on the solubility of TCM active components in water and ethanol. Partial solubility data are shown in Table 1. In general, the solubility of moderately polar active components in ethanol is generally higher than the solubility in water, indicating that these components theoretically will not precipitate during EPP.

**Table 1:** Solubility of some TCM active components in water and ethanol

Component category	Active component	Solubility in water	Solubility in ethanol	Unit of solubility	Temperature/ °C	References
Phenolic acids	Rosmarinic acid	1.35 × 10^−2^	2.68 × 10^−1^	mol/mol	20	[11]
	Gallic acid	1.07	23.7	g/100 g	25	[12]
	Gentisic acid	2.20	45.5	g/100 g	25	[12]
Phenols	2-Naphthol	0.00	2.49 × 10^−1^	mol/mol	20	[13]
	Catechol	7.52 × 10^−2^	3.57 × 10^−1^	mol/mol	20	[13]
	Hydroquinone	1.02 × 10^−2^	1.88 × 10^−1^	mol/mol	20	[13]
	Curcumin	2.15 × 10^−8^	6.62 × 10^−4^	mol/mol	20	[14]
	*trans*-Resveratrol	2.90 × 10^−6^	1.56 × 10^−2^	mol/mol	20	[15]
Flavonoids	Apigenin	7.00 × 10^−7^	2.44 × 10^−4^	mol/mol	15	[16]
	Baicalein	6.63 × 10^−6^	1.04 × 10^−3^	mol/mol	20	[17]
	Chrysin	1.26 × 10^−5^	6.89 × 10^−3^	mol/mol	20	[18]
	Genistein	5.30 × 10^−6^	3.74 × 10^−2^	mol/L	25	[19]
	Luteolin	1.75 × 10^−6^	1.88 × 10^−3^	mol/mol	25	[20]
	Hesperetin	2.40 × 10^−6^	7.30 × 10^−2^	mol/L	20	[21]
	Hesperidin	1.42 × 10^−7^	3.08 × 10^−5^	mol/mol	20	[22]
	Naringenin	6.62 × 10^−7^	9.20 × 10^−3^	mol/mol	20	[23]
	Naringin	9.76 × 10^−7^	3.98 × 10^−5^	mol/mol	20	[24]
	Daidzin	4.10 × 10^−6^	3.97 × 10^−4^	mol/mol	20	[25]
	Daidzein	6.08 × 10^−8^	2.70 × 10^−4^	mol/mol	20	[26]
Alkaloids	Piperine	1.07 × 10^−5^	8.99 × 10^−3^	mol/mol	25	[27]
Coumarins	Osthole	4.86 × 10^−7^	1.75 × 10^−2^	mol/mol	20	[28]
	Isoimperatorin	7.42 × 10^−7^	3.77 × 10^−3^	mol/mol	20	[29]

**Fig. 1:**
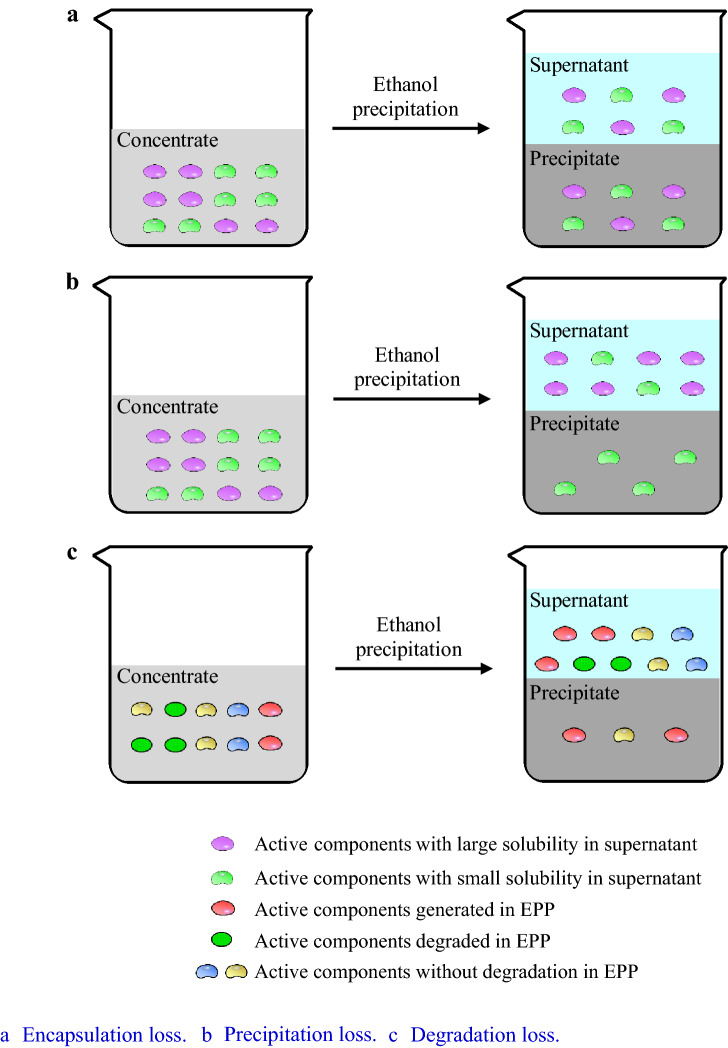
Loss mechanism of active components

However, the loss of active components during EPP has been observed by many researchers [30]. The authors believe that there are at least three reasons for the loss of active components, including encapsulation loss, precipitation loss, and degradation loss (Fig. 1). During EPP, the encapsulation loss can result from incomplete mixing of the ethanol and the concentrate, which can result in agglomeration and liquid encapsulation. Therefore, the coating phenomenon results in part of the active components not being dissolved in ethanol, leading to partial loss. The insufficient contact between ethanol and concentrate is caused by the large density difference between these solutions, the large viscosity of the concentrate, and the large amount of precipitate produced during EPP. Concentrate with higher dry matter content probably led to more encapsulation loss of active components [31]. The encapsulation loss is greatly affected by concentrate properties, EP equipment and operation conditions, and it will be reduced after a long standing time.

When the author studied the EPP of Danshen Chuanxiong mixed decoction concentrate [32], it was found that the total content of danshensu and salvianolic acid B in the supernatant and precipitation after EPP was less than the total amount in the concentrate. However, the content of lithospermic acid in the supernatant and precipitation was significantly higher than that in the concentrate. This phenomenon indicates that the active components may be degraded or polymerized in the supernatant, resulting in degradation loss.

The dissociation constant values of salvianolic acids in several liquid–liquid equilibrium systems were determined [33], which verified that phenolic acids often exist in medicinal plants in the form of phenolic acid salts according to the pH value of concentrate. The solubility of phenolic acid salts in the supernatant is usually lower than that of phenolic acid molecules, which is the reason for the precipitation loss of phenolic acids.

Three types of active component loss may exist simultaneously in an EPP. Degradation loss can be determined by comparing the total amount of an active component before and after EPP. For an active component with large solubility in the mixed solvent of water and ethanol, encapsulation loss probably exists when some of this active component is found in the precipitate. However, it is still difficult to distinguish between precipitation loss and encapsulation loss when the solubility of the active component is not very large.

## EPP application for bioactive component purification

EPP is a conventional purification technology for bioactive components in TCMs, such as alkaloids, flavonoids [30], anthraquinones [34], organic acids, polysaccharides, and proteins. Polysaccharides and proteins are usually collected from the precipitate of EPP. The polysaccharides of TCMs may possess antioxidant activity [35, 36], anti-tumor activity [37], immunomodulatory effects [38], and hepatoprotective effect [39]. By adjusting the ethanol concentration in supernatant, polysaccharides with different molecular weight distributions can be obtained. The general rule is that higher ethanol concentration in supernatant results in the precipitation of polysaccharides with smaller molecular weights. Therefore, EPP is also widely used in the grading of polysaccharides [40]. Alkaloids, flavonoids, organic acids, saponins, or other active components of TCMs are usually enriched in the supernatant after EPP. At most occasions, a mixture of these active components and other components is prepared. Therefore, EPP is used to prepare the so-called “total alkaloids”, “total flavonoids”, “total phenolic acids”, or “total saponins”. EPP is also reported in the precipitation of plant DNA [41], especially in the DNA barcoding identification of Chinese medicinal materials.

## EP and TCM quality

In order to ensure the safety and effectiveness of TCMs, pharmacodynamic indices are widely used in the research of the manufacturing processes of TCMs. Compared with physical and chemical indices, pharmacodynamic indices can reflect the efficacy as a whole for TCMs. Some works on the relationship between EPP and pharmacodynamic indices are listed in Table 2. There are more than ten pharmacodynamic indices were reported in the evaluation of EP products, such as analgesic effect, anti-hypertensive effect, antipyretic, anti-inflammator, and so on.

**Table 2:** Relationship between EPP and pharmacodynamic indices

Medicinal materials or compound preparations	The pharmacodynamic index changes after EPP	References
Wubie granule intermediates	No significant difference	[42]
Yanshuning compound	No significant difference	[43]
Zhuang Medicine Baijin granules	No significant difference	[44]
Dingtongning granules	No significant difference	[45]
Changkang granules	No significant difference	[46]
Eryan Huguo decoction	No significant difference	[47]
Xikebao decoction	No significant difference	[48]
Shenqi Sherong pills	No significant difference	[49]
Gualou-Xiebai extracts	Antioxidant activity was enhanced	[50]
Lidan Paidu prescription	No significant difference	[51]
*Prunella vulgari*	Anti-hypertensive effect was enhanced	[52]
Scutellariae radix extract	After EP twice, antipyretic and anti-inflammatory effects were weakened compared with EP once	[53]
*Chimonobambusa quadrangularis*	Antioxidant activity was enhanced	[54]
Guizhi Zhumian capsule	Sleep improvement function was reduced	[55]

Many researchers found that pharmacodynamic indices changed little after EPP. It indicated that EPP could probably reduce daily dosage of the preparation without lowering its efficacy. Drug efficacy was significantly enhanced after EPP in some published works [50, 52, 54]. It means that active components were enriched after EPP. Some researchers found that pharmacodynamic indices remarkably improved when the apparent content of ethanol reached about 75% [50, 54]. However, Du et al. observed that its effects of improving sleep are weakened after EPP [55]. The possible reason was that some active components lost in EPP because of precipitation, degradation, or encapsulation. The active components lost in EPP may possess a direct or synergistic drug efficacy.

Overall, most works showed that EPP can reduce daily dosage of TCM preparation without significantly lower drug efficacy. However, EPP is not suitable for some TCMs. Ethanol content in EP supernatant should be optimized for keeping or enhancing drug efficacy.

## EP equipment

**Fig. 2:**
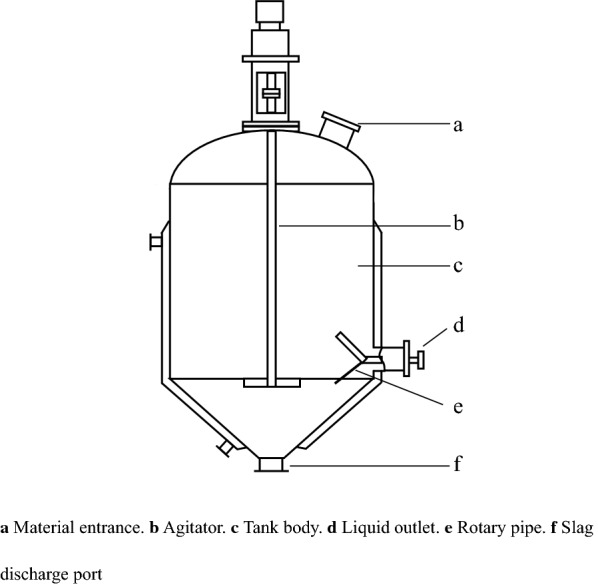
Schematic diagram of EP equipment with mechanical agitation

EPP is commonly carried out in an EP tank in the industry, and its schematic diagram is shown in Fig. 2. The concentrate and ethanol in the EP tank can be mixed either by mechanical agitation or air agitation; the former is widely used [56]. The advantage of air agitation in an EP tank is that there are no moving parts in the tank, and the possibility of equipment failure is slight. The disadvantage is that the air will cause evaporation and loss of ethanol [57]. If the EP tank is provided with a jacket, it can be cooled by refrigerating with low-temperature liquid. If the EP tank is not provided with a jacket, it can be moved into a refrigerated room for refrigeration. After the EP supernatant is collected, the EP precipitation is discharged from the slag outlet.

**Fig. 3:**
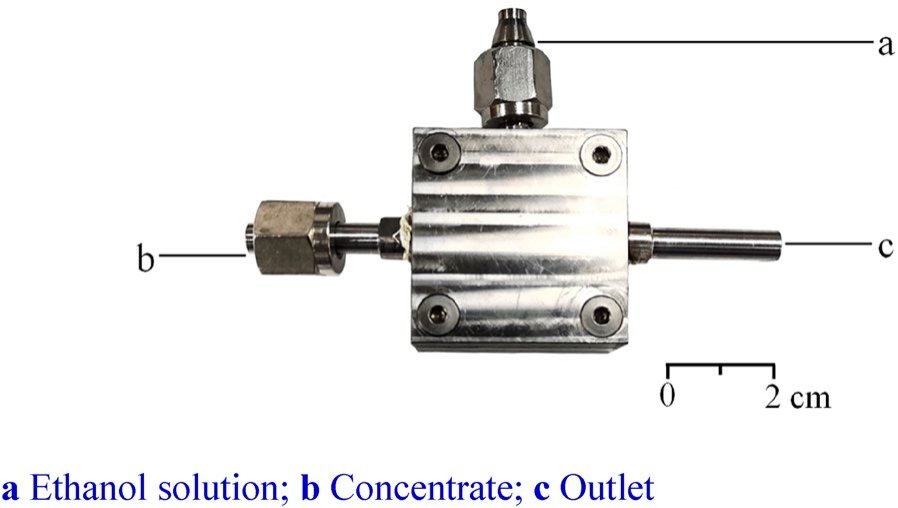
The appearance of EP micromixer

At present, the main improvement directions of EP equipment are to improve the mixing effect of the concentrate and ethanol and to reduce the difficulty of slagging after EPP. The authors used a micromixer to continuously mix the concentrate and ethanol to achieve a continuous steady-state process during the ethanol addition process (Fig. 3) [58]. This method can be used to control the amount of ethanol addition by adjusting the flowrates of ethanol and concentrate. The encapsulation loss of active components were also effectively reduced [31]. In conventional equipment, ethanol is added slowly and stirred quickly to reduce encapsulation loss. For the micromixer, the faster the ethanol is added, the better the mixing effect, and time can be saved. Yu et al. [59] used a pressure-type mechanical atomization device to atomize the concentrate and ethanol to improve mixing effect. Changing the position of the agitator or improving the structure of the paddle can also improve the mixing effect [60, 61]. Adding a shear agitator and a slag outlet at the bottom of the EP tank can reduce manually clean precipitation and improve the efficiency of slag removal [62]. These improved EP equipment will be more widely used in the future.

## Critical parameters and optimization methods of the EPP

Table 3 lists the experimental design methods, optimization goals, and critical factors, based on more than 70 studies reported about the EPP in the past 10 years. Researchers mainly use single factor design, orthogonal design, fractional factorial design, and Plackett–Burman design to determine the critical factors of EPP. Compared with single factor design and orthogonal design, fractional factorial design and Plackett–Burman design can use only a few experiments to explore the influence of many parameters.

**Table 3:** The experimental design, optimization objective, and critical factors of the EPP

Medicinal materials or compound preparations	Experimental design	Optimization objective	Critical factors	References
Danshen	Plackett–Burman design, Box–Behnken design	Highest protocatechualdehyde content	ρ, pH value of EP solution	[63]
Danshen	Orthogonal design	The highest comprehensive score of impurity removal and retention rate of total salvianolic acids	ρ, C_e_, ethanol flowrate	[64]
Danshen	Central composite design	The highest purity of danshensu	WC_c_, C_e_, DE	[65]
Cinobufacini extract	Single factor design, orthogonal design	The highest comprehensive score of cinobufacini retention, impurity removal rate and cinobufacini purity	Φ, ρ, C_e_, T	[66]
Kuanxin oral liquid	Orthogonal design	Low dry extract rate or high danshensu content	ρ, Φ	[67]
Danshen	Fractional factorial design, Box–Behnken design	The highest removal of saccharides	SC_c_, DE, T, hydrochloric acid content	[68]
Danhong injection	Fractional factorial design	Not optimized	WC_c_, C_e_, DE	[69]
Danhong Injection	Box–Behnken design	Content of five active components such as danshensu and total solids were within the control range	WC_c_, C_e_, DE	[70]
Danshen	Box–Behnken design	Not optimized	ρ, DE, T	[71]
Schisandrae Chinensis fructus	Central composite design	Not optimized	DE, C_e_	[72]
Guanxinning injection	Box–Behnken design	Constructed a satisfaction function with the retention rate of active components such as danshensu and total solids removal rate to maximize the value	SC_c_, DE, T	[73]
Danhong injection	Central composite design	Retention rate and total solids removal rate of danshensu and other active components meet the standards	WC_c_, C_e_, DE, T	[74]
Qingmai granules	Orthogonal design	High dry extract rate or high diosgenin content	ρ, Φ	[75]
Gardeniae FRUCTUS	Orthogonal design	The highest comprehensive score of peak area of geniposide and total peak areas of fourteen chemical components	ρ	[76]
Hemorrhoid fumigants	Orthogonal design	The highest comprehensive score of total alkaloid content and dry extract rate	Φ	[77]
Fermentative fluid of Cordyceps	Orthogonal design	The highest extracellular polysaccharides content	DE	[78]
Safflower injection	Orthogonal design	The highest comprehensive score of total flavonoid loss rate and dry extract rate	t	[79]
Changkang granules	Single factor design	The highest comprehensive score of impurity removal rate and content of four active components such as hypericin	ρ, Φ	[46]
Ephedran	Single factor design, central composite design	The highest polysaccharide yield	C_c_, Φ	[80]
Lanshen lipid-lowering prescription	Orthogonal design	High ratio of total saponin content to dry extract rate	t	[81]
Fufang Shenqi soft capsules	Orthogonal design	High total polysaccharide content and low dry extract rate	ρ, Φ	[82]
Dangshen	Plackett–Burman design, Box–Behnken design	Total flavonoid recovery, dry matter removal, and pigment removal meet the standards	SC_c_, C_e_, DE	[83]
Liuwei Dihuang decoction	Plackett–Burman design, Box–Behnken design	The highest transfer rates of morroniside, loganin, and paeoniflorin	ρ, Φ, t, SS, centrifuge or not	[84]
*Trillium tschonoskii* maxim	Single factor design, orthogonal design	The highest polysaccharide yield	Φ	[85]
Liuwei Dihuang decoction metabolized by photosynthetic bacteria	Orthogonal design	The highest comprehensive score of paeonol content, polysaccharide content, and dry extract rate	C_c_	[86]
Qiguiyin formula	Single factor design	The highest comprehensive score of astragaloside content, chlorogenic acid content, and dry extract rate	C_c_, Φ	[87]
*Lonicerae Japonicae* and *Artemisiae Annuae Herba* in reducing injection	Single factor design, Box–Behnken design	Constructed a satisfaction function based on a comprehensive score of the transfer rate of five components such as neochlorogenic acid and solid content to maximize the value	ρ, temperature before EP, T	[88]
Bishuang Paidu granules	Single factor design	The retention rate of baicalin and decrement of solid matter were high	Φ	[89]
Herba Sarcandrae	Single factor design, orthogonal design	The highest transfer rate of tannin	Stirring time, T	[90]
*Zizyphus jujube* cv. Dongzao	Orthogonal design	The highest polysaccharide yield	CR, DE	[91]
Zhenjing Xiehuo granules	Orthogonal design	The dry extract rate, liquiritin content, and salvianolic acid B content were highest	Φ	[92]
Xuanbi Antong formula	Orthogonal design	The highest content of five components such as salvianolic acid B and dry extract content	No significant factor	[93]
*Meretrix meretrix*	Orthogonal design	The highest comprehensive score of yield and mass fraction of water-soluble polysaccharide	CR, Φ	[94]
Guben Bushen oral liquid	Orthogonal design	The highest polysaccharides yield	Φ	[95]
Tongfengxiao granules	Orthogonal design	The highest comprehensive score of the content of berberine hydrochloride and salvianolic acid B	t	[96]
Zhimahuang group in Shufeng Dingchuan granules	Box–Behnken design	The highest comprehensive score of transfer rates of ephedrine hydrochloride, pseudoephedrine hydrochloride, amygdalin, and solid content	ρ, Φ, t	[97]
Shenqi compound recipe	Box–Behnken design	Constructed a satisfaction function with polysaccharide content and dry extract rate to maximize the value	Φ, t, CR	[98]
Wubie granules intermediates	Single factor design	Transfer rates of stilbene glucoside, asperosaponin VI, and solid content were high	ρ, Φ, t	[42]
Qifang Bimin granules	Single factor design	The highest comprehensive score of extraction rates of astragaloside IV and paeoniflorin	ρ	[99]
Bazhen granules	Orthogonal design	Dry extract rate and paeoniflorin content were high	Φ, t	[100]
Qianyang Yuyin granules	Orthogonal design	The highest comprehensive score of stilbene glycoside content and dry extract rate	No significant factor	[101]
Biqiu granules	Plackett–Burman design, Box–Behnken design	The highest comprehensive score of caffeic acid content and rosmarinic acid content	ρ, Φ, t	[102]
Chailing Hugan granules	Orthogonal design	The highest comprehensive score of dry extract rate, total flavonoids content, and polysaccharides content	t, C_c_	[103]
Dendrobium Candidum eye drops	Orthogonal design	The highest crude polysaccharides content	No significant factor	[104]
Ganmaoling granules	Single factor design	The highest dry extract rate	C_e_, ρ, Φ, t	[105]
Majiezhike granules	Orthogonal design	The highest comprehensive score of retention rate of ephedrine hydrochloride and decrement of solid matter	t	[106]
Shouwu Granula	Orthogonal design	The highest comprehensive score of stilbenes content and dry extract rate	No significant factor	[107]
Lidan Paidu prescription	Orthogonal design	The highest comprehensive score of extraction rate and content of chlorogenic acid, jasminoidin, and salvianolic acid B	ρ	[51]
Qingyan Shuanghou granules	Box–Behnken design	Constructed a satisfaction function with extraction rate and chlorogenic acid extraction yield to maximize the value	ρ, Φ, t	[108]
Liqifuwei oral liquid	Orthogonal design	The highest of anthraquinone transfer and dry extract rate	ρ, Φ	[109]
Tong Fengqing cataplasm	Orthogonal design	The highest of total retention of matrine and oxymatrine	ρ	[110]
Dingtongning granule	Orthogonal design	The highest comprehensive score of dry extract rate and transfer rates of paeoniflorin and ferulic acid	ρ, Φ	[45]
Zhidanhuayu formula	Single factor design	The highest comprehensive score of dry extract rate, paeoniflorin content, and astragaloside IV content	ρ, Φ	[111]
Chaixiong mixture	Single factor design	The highest comprehensive score of total saikosaponin content and impurity removal rate	ρ, Φ	[112]
Poria Cocos	Single factor design, orthogonal design	The highest yield of water-insoluble polysaccharide	CR, Φ, t	[113]
Dibutyl particles	Orthogonal design	The highest phenanthrene content and dry extract rate	C_c_, Φ	[114]
Xuanfei Zhike granule	Orthogonal design	The highest comprehensive score of hesperidin content, tectoridin content, and dry extract rate	No significant factor	[115]
Compound Cornu Cervi Degelatinatum	Orthogonal design	The highest content of monotropein, loganin and chiratin, and dry extract rate	Φ	[116]
*Crataegus pinnatifida*	Plackett–Burman design, Box–Behnken design	The highest retention rate of total flavonoids	SS, DE, ρ	[117]
Qizhi Yifei granules	Single factor design	The high extraction rate of astragaloside, quercetin-3-*O*-β-_d_-glucose-7-*O*-β-_D_-gentian diglucoside and dry extract rate	ρ, Φ	[118]
Qingyan oral liquid	Orthogonal design	The highest comprehensive score of the transfer rate of irisflorentin and total glycosides	Φ	[119]
Yinchen mixture	Single factor design, central composite design	Constructed a satisfaction function with transfer rates of geniposide and solid removal rate to maximize the value	ρ, Φ, SS	[120]
Shiwei Ehuang granules	Orthogonal design	The highest astragaloside A content	ρ, Φ	[121]
Webikang granules	Orthogonal design	The highest comprehensive score of hesperidin content and dry extract rate	No significant factor	[122]
Panax Ginseng and Pueraria Lobata concentrated decoction	Box–Behnken design	The high precipitation rate and total saponin content	Φ, t	[123]
Compound lipid-lowering Sustained-release tablets	Single factor design, orthogonal design	The highest comprehensive score of extraction rates of salvianolic acid B, nuciferine and total flavonoids	ρ	[124]
*Sophora flavescens*	Box–Behnken design	The highest comprehensive score of total alkaloid extraction rate and dry extract rate	Φ	[125]
A Formula including radix Puerariae, Radix Paeoniae Rubra, Desertliving Cistanche, and Pinellia tuber	Orthogonal design	The highest comprehensive score of the content of paeoniflorin, puerarin, echinacoside, and dry extract content	ρ	[126]
Kangzhi Suppository	Orthogonal design	The highest comprehensive score of berberine hydrochloride content and paste-forming rate	ρ	[127]
Fufang Roucongrong Mixture	Orthogonal design	The highest comprehensive score of retention amounts of verbascoside, lobetyolin, and salvianolic acid B	SS, ρ, Φ	[128]
Fufang Shuanghua oral liquid	Orthogonal design	The highest comprehensive score of (*R, S*)-goitrin retention rate, precipitation rate of protein/polysaccharide/tannin, and solid content	t	[129]
Astragali radix	Plackett–Burman design	Not optimized	Φ, T	[130]

The frequency of each critical factor in Table 3 is listed in Table 4. Several factors, such as the density, concentration ratio, concentration, water content, and solid content of the concentrate, can reflect the amount ratio between the solid and solvent in the concentrate. Lower solvent content of concentrate, higher amount of ethanol, and higher concentration of ethanol all lead to a higher ethanol content of the EP supernatant. The ethanol content of EP supernatant affects the solubility of the components in the concentrate, so the above factors are often critical. The standing temperature is also often considered a critical parameter, mainly because temperature affects solubility. In a few pieces of literature, the stirring speed and ethanol flowrate are also considered critical parameters. From the above mechanism analysis, it can be seen that when the concentration of concentrate is high, it is difficult to mix the ethanol and concentrate completely. A high stirring speed or slow ethanol flow rate is favorable for mixing, so it may also become a critical parameter. Many researchers have found that the standing time also has a significant effect on the effect of EPP, probably because the concentrate encapsulated in the precipitate can gradually dissolve after standing for a long time, thereby changing the composition of the supernatant.

**Table 4:** The frequency of critical factors

No.	Critical factors	Research frequency
1	Φ	36
2	ρ	31
3	t	16
4	DE	12
5	C_e_	9
6	T	8
7	C_c_	5
8	CR	4
9	SS	4
10	WC_c_	4
11	SC_c_	3
12	Others	6

**Fig. 4:**
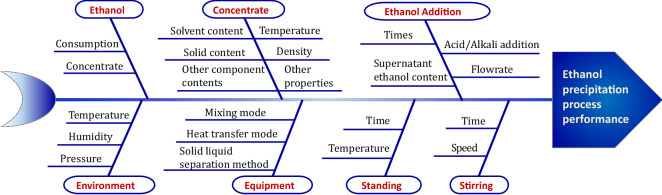
Ishikawa diagram analysis for EPP

According to the researches shown in Table 3, the Ishikawa diagram of the EPP was sorted out, as shown in Fig. 4. This diagram involves many factors, such as ethanol, concentrate, ethanol addition, environment, equipment, standing, and stirring.

Many studies have considered the density, water content, and solid content of the concentrate. However, the differences between different batches of concentrate are mainly reflected in the content of each component in the total solid and other physical and chemical properties except density. There are few studies on these factors. Zhang et al. [131] screened out the critical properties of the concentrate of the first EP of Danshen injection by stepwise regression and partial least square method. The results showed that the pH and caffeic acid content of the concentrate are the critical parameters affecting the phenolic acid content in the supernatant of the second EPP. Furthermore, Yan et al. [132] found that the retention rate of phenolic acids in the second EPP of Guanxinning injection was mainly affected by the contents of danshensu, caffeic acid, and salvianolic acid B in the concentrated supernatant of the first EPP. These research findings further indicate that the quality of the supernatant is affected by the properties of EP raw materials.

There are many process parameters or concentrate properties that may affect EP results. However, the parameters of EPP are usually limited in narrow ranges in industry. Therefore, the quality control of concentrates is very important to keep batch-to-batch consistency of supernatants. By improving the quality control of decoction pieces and upstream processes of EPP, the quality of concentrate can be controlled within a proper range to promote the quality of the EPP.

## Modeling and calculation of the EPP

### Semimechanical modeling

Assuming that the concentrate is composed of water and total solids, there is no water in EP precipitation, and the mass fraction (*ϕ*) of ethanol in the supernatant solvent is defined in Eq. (1) [73]:

1 $$\phi = \frac{{EC_{e} \times ECR }}{{WC_{c} + ECR }}$$

where *EC*_*e*_ refers to the mass fraction of ethanol used in EPP, *ECR* refers to the mass ratio of ethanol and the concentrate, and *WC*_*c*_ refers to the water content of the concentrate. The content of saturated components in the supernatant is also their solubility (*S*), which is defined in Eq. (2) [32]:

2 $$S = S_{w} \times \left( {1 - \phi } \right)^{\alpha }$$

where *S*_*w*_ refers to the solubility of the component in pure water, and *α* refers to the parameter to be fitted.

Organic acids may exist in EP systems in molecular form and salt form. If precipitation loss occurs, it may be because the organic acid salts are saturated in the supernatant. The relationship between the *pK*_*a*_ value, the solubility of the organic acid salt (*S*_*A*_), the *pH* of the supernatant, the total concentration (*C*_*A*_) of the organic acid and organic acid salts in the supernatant is shown in Eq. (3), which can also be used to calibrate the *pKa* value of phenolic acid and solubility of phenolic acid salts [32].

3 $$\frac{{C_{A} }}{{S_{A} }} = 10^{{\left( {pK_{a} - pH} \right)}} + 1$$

Till now, the mechanism research of EPP is not in-depth. Accordingly, the current semimechanical models are relatively simple. There are no reports on mechanism model of EPP.

### Statistical modeling

Single factor design, orthogonal design, central composite design, and Box–Behnken design are often used to optimize the parameters of the EPP. Compared with single factor design, response surface design can consider the interaction between factors. With the same number of factors, response surface design has more experiments than orthogonal design. Still, after modeling, response surface design can obtain the optimal global condition in the research scope. Central composite design and Box–Behnken design usually adopt polynomial modeling after obtaining the experimental data. The form is as follows Eq. (4):

4 $$Y = b_{0} + \sum\nolimits_{{i = 1}}^{m} {b_{i} X_{i} } + \sum\nolimits_{{i = 1}}^{m} {b_{{ii}} X_{i}^{2} } + \sum\nolimits_{{i = 1}}^{{m - 1}} {\sum\nolimits_{{i = i + 1}}^{m} {b_{{ij}} X_{i} X_{j} } } ~$$

where *Y* refers to the evaluation index of the EPP, *b*_0_ refers to a constant term, *b*_i_, *b*_ii_, and *b*_ij_ refer to regression coefficients, and *m* refers to the number of factors in the experimental design. The quality of the EPP can be evaluated by the index component content, component retention rate, impurity removal rate, total solid removal rate, and pharmacodynamic index [102]. Modeling can be simplified by using methods such as stepwise regression. Polynomial models are easy to build and explain. However, the models are difficult to be extended to another EP equipment or another batch of concentrate.

### Parameter optimization calculation

The largest multi-index comprehensive score, largest satisfaction function value, and all indexes falling within the preset ranges are commonly used optimization objectives. The multi-index comprehensive scoring method and the satisfaction function can be used to address different dimensions of process evaluation indicators, but the subjectivity is large when determining weights. When there is a strong correlation between the evaluation indexes of the EPP, the use of the satisfaction function should be carefully performed [133]. A group of optimal parameter combinations is often obtained to maximize the comprehensive score of multiple indicators or the satisfaction function. Nevertheless, this approach is not conducive to flexible adjustment of process parameters in the actual production of multiple batches.

When using all the indexes that fall into the preset ranges as the parameter optimization target, the optimized process parameter ranges can be calculated generally. This research method is in line with the design space concept of quality by design (QbD) [134]. The parameter variation within the design space is not considered as a process change, so the approach is beneficial to pharmaceutical companies not only to increase production flexibility but also to reduce unnecessary supervision. The optimal parameter range can be obtained by using the overlapping method and the probability-based method [135]. The probability-based method quantifies the assurance of EPP quality with probability values in the optimization of parameter ranges. The probability values calculated by the experimental error simulation method [135] and the parameter disturbance simulation method [136] are more accurate.

Yan et al. established a mathematical model between the contents of active components in concentrates, the process parameters, and the properties of supernatant by adopting a feedforward control strategy. Then, according to the contents of active components in the concentrate, the parameters of the EPP of Danhong injection were adjusted. This method can improve the consistency of the active component contents in the supernatant [70]. The authors noticed that the refrigeration temperature for EP in the production of pharmaceutical companies is affected by the season. Therefore, it is proposed to set the refrigeration temperature as the noise parameter and optimize the range of other easily controlled parameters to reduce the impact of noise parameter fluctuation [74].

Operating process parameters with design space, varying process parameters according to the change of concentrate quality, or adjusting controllable parameters to lower the effects of noise parameters can all improve the batch-to-batch consistency of supernatant quality after EPP.

### The monitoring method of the EPP

In the production of TCM, an ethanol meter is widely used to detect the apparent ethanol content of the supernatants on the spot. This method is simple and practical, but only the density information of the liquid can be obtained.

The monitoring technology and indicators of EPP in the literature are listed in Table 5. At present, near-infrared spectroscopy (NIR) is widely used due to its simple sample preprocessing, fast speed, losslessness, large amount of information collected, etc. In general, partial least square regression and other methods can be used to correlate the NIR information with the contents of index components/major components in the supernatant. Spectral preprocessing methods have a great influence on the modeling results. Common preprocessing methods include standard normal variate, multiplicative scatter correction, Savitzky-Golay smoothing, Norris-Williams smoothing, first derivative, second derivative, etc. By establishing a multivariate statistical process control model, the control limit of the process operation statistics (such as Hotelling T^2^, squared prediction error, and principal component score) is set up, and the process trajectory diagram is drawn. The multivariate statistical process control model can monitor the EPP in real-time and sensitively judge the normal operation state of the process. The establishment of a multivariate statistical process control model is helpful further to implement the feedback control of the EPP.

**Table 5:** The monitoring technology and process indicators of EP

Medicinal materials or compound preparations	Detection technology	Monitoring indexes	References
Danshen	NIR	The concentration of danshensu and protocatechualdehyde, and total solid content	[137]
Danshen	NIR	The concentration of six active components such as danshensu, solid content, scores of the first principal component, Hotelling T^2^, squared prediction error	[138]
Rukuaxiaopian	NIR	The concentration of danshensu and hesperidin	[139]
Danshen	NIR	Tannin concentration	[140]
Danshen	NIR	Scores of the first principal component, Hotelling T^2^, squared prediction error	[141]
Cinobufacini	NIR	Indole alkaloids concentration	[142]
*Lonicerae Japonicae*	NIR	Hotelling T^2^, squared prediction error	[143]
*Lonicerae Japonicae*	NIR	Chlorogenic acid concentration	[144]
*Lonicerae Japonicae*	NIR	Chlorogenic acid concentration	[145]
Danhong Injection	NIR	Solid content and concentration of five active components such as danshensu	[146]
Reduning Injection	NIR	The concentration of four active components such as neochlorogenic acid	[147]
Reduning Injection	NIR	The concentration of chlorogenic acid and solid content	[148]
Shenzhiling Oral Solution	NIR	The concentration of six active components such as paeoniflorin	[149]
Dangshen	NIR	The concentration of lobetyolin, total flavonoids, pigments, and total solid contents	[150]
Astragali Radix	NIR	Scores of the first principal component, Hotelling T^2^, squared prediction error, the concentration of six active components, and total solid content	[151]
Danshen	NIR	The concentration of glucose, fructose, and sucrose	[152]

In general, spectrum of EP system is rich in information. The process monitoring method based on spectrum can not only judge the process state, but also quantify the concentrations of specific components in combination with chemometrics.

## Conclusions and perspective

Based on the extensive review, great progress has been made in the study of process parameters, optimization methods, and process monitoring methods of EP of TCM. Problems still exist in industrial EP, including the loss of active components, the long time necessary for refrigeration, the quality difference between batches of EP supernatants, etc. In the future, EP technology research can be carried out from the following directions:

### Further study on the mechanism of EP

The difference in concentrates between batches is mainly reflected in the fluctuation of the content of the components. At present, there have been reports about the influence of ethanol content in the supernatant on the solubility of Chinese herbal medicinal components. Nevertheless, there is no study on the influence of the content of Chinese herbal medicinal components on the solubility of other components. Therefore, it is not yet possible to describe the effect of the composition change of concentrate on the effect of EP. It is also difficult to accurately predict the material transfer and drug delivery rule of EPP.

### Further study on the effects of EP on TCM quality

EPP is widely used in TCM industry from the last century. However, the quantitative effects of EPP on TCM quality are still unclear. The relationship between TCM substances and its quality is generally nonlinear. Therefore, some newly developed artificial intelligence technology can probably be used for the investigation of EPP and TCM quality. For example, as a typical algorithm of deep learning, convolutional neural network (CNN) can be a useful tool to deal with nonlinear quantitative problems [153, 154].

### Establish a stricter quality control method for concentrates

At present, the concentrate quality in the industry is mostly controlled by density or volume. However, less attention has been paid to the chemical composition of the concentrate. It is recommended that the concentrate be used as one of the critical intermediates, and the quality standard of its composition should be set. Yan et al. used the quantitative model of process parameters, raw material properties, and EP evaluation index to back-calculate the quality standard of a concentrate [155]. This work provides a scientifically based method to set the quality standard of the concentrates. Where permitted by regulations, it can be considered that EP can be carried out after a mixed concentrate is prepared with different batch concentrates, which will help to improve the consistency of the components of the supernatant.

### Enrich the detection technology of the EPP

NIR combined with multivariate statistical analysis is used to detect indicators/major components or to detect process trajectories. NIR has many advantages, but the equipment cost is high, and the renewal and maintenance of the multivariate statistical model require professionals. In addition, there is still no means to detect the amount of encapsulation loss. Therefore, it is still necessary to develop simpler and easier-to-use detection technology.

### Develop high-efficiency digital ethanol precipitation equipment

At present, the structure of EP equipment is simple, and process control relies heavily on manual work. The energy and material consumption are still high. Therefore, a complete set of intelligent EP equipment should be developed based on multidisciplinary technology. This equipment should be able to improve the efficiency of heat and mass transfer, quickly collect and analyze process data, and realize the automatic control of EPP.

## Data Availability

All reported or analyzed data in this review are extracted from published articles.

## Abbreviations

TCM: Traditional Chinese medicine
EP: Ethanol precipitation
EPP: Ethanol precipitation process
NIR: Near-infrared spectroscopy
ρ: Density of concentrate
C_c_: Concentration of concentrate
SC_c_: Solid content of concentrated supernatant
WC_c_: Water content of concentrated extract
DE: Ethanol consumption
C_e_: Ethanol concentration
Φ: Ethanol content of ethanol precipitate
T: Ethanol precipitation temperature
t: Ethanol precipitation time
SS: Stirring speed
CR: Concentration ratio
